# *Pseudomonas aeruginosa* PAO1 Is Attracted to Bovine Bile in a Novel, Cystic Fibrosis-Derived Bronchial Epithelial Cell Model

**DOI:** 10.3390/microorganisms10040716

**Published:** 2022-03-26

**Authors:** Shekooh Behroozian, Inmaculada Sampedro, Basanta Dhodary, Stephanie Her, Qianru Yu, Bruce A. Stanton, Jane E. Hill

**Affiliations:** 1 Department of Chemical and Biological Engineering, University of British Columbia, 2360 E Mall, Vancouver, BC V6T 1Z3, Canada; shekooh@mail.ubc.ca (S.B.); basanta.dhodary@ubc.ca (B.D.); 2 Department of Microbiology, Faculty of Pharmacy, University of Granada, Campus de Cartuja s/n, 18071 Granada, GR, Spain; isampedro@ugr.es; 3 Biomedical Research Center (CIBM), Biotechnology Institute, Avda del Conocimiento s/n, 18100 Armilla, Granada, GR, Spain; 4 Thayer School of Engineering, Dartmouth College, Department of Microbiology and Immunology, Geisel School of Medicine at Dartmouth, Hanover, NH 03755, USA; stephanie.cindy.her@gmail.com (S.H.); qianru.yu@jax.org (Q.Y.); bruce.a.stanton@dartmouth.edu (B.A.S.)

**Keywords:** chemotaxis, bile, *Pseudomonas aeruginosa*, cystic fibrosis, bronchial epithelial cells

## Abstract

Cystic fibrosis (CF) is a life-threatening, inherited, multi-organ disease that renders patients susceptible throughout their lives to chronic and ultimately deteriorating protracted pulmonary infections. Those infections are dominated in adulthood by the opportunistic pathogen, *Pseudomonas aeruginosa* (*Pa*). As with other advancing respiratory illnesses, people with CF (pwCF) also frequently suffer from gastroesophageal reflux disease (GERD), including bile aspiration into the lung. GERD is a major co-morbidity factor in pwCF, with a reported prevalence of 35–81% in affected individuals. Bile is associated with the early acquisition of *Pa* in CF patients and in vitro studies show that it causes *Pa* to adopt a chronic lifestyle. We hypothesized that *Pa* is chemoattracted to bile in the lung environment. To evaluate, we developed a novel chemotaxis experimental system mimicking the lung environment using CF-derived bronchial epithelial (CFBE) cells which allowed for the evaluation of *Pa* (strain PAO1) chemotaxis in a physiological scenario superior to the standard in vitro systems. We performed qualitative and quantitative chemotaxis tests using this new experimental system, and microcapillary assays to demonstrate that bovine bile is a chemoattractant for *Pa* and is positively correlated with bile concentration. These results further buttress the hypothesis that bile likely contributes to the colonization and pathogenesis of *Pa* in the lung, particularly in pwCF.

## 1. Introduction

Bacterial chemotaxis is the directional navigation of an organism toward or away from certain chemical gradients, which may enhance their access to favorable substances or avoid hostile niches, respectively [1,2,3]. Chemotaxis signaling pathways are broadly distributed among bacterial pathogens and the phenotype is linked to their pathogenicity, playing an essential role in initial localization, subsequent colonization, and infection of the host [4]. For decades, the vast majority of bacterial chemotaxis studies have been performed using in vitro capillary assay methods, initially developed by Adler (1966) [1] for the quantification of *Escherichia coli* chemotaxis, or with some further modifications for other bacterial pathogens [5]. To better mimic the in situ bacterial chemoattractions during pathogenesis, we have developed a model that builds on the principles of Adler’s classic assay system. Our new chemotaxis experimental system utilizes a human cystic fibrosis (CF) bronchial epithelial (CFBE) cell environment that mimics CF airway epithelia [6,7,8]. Here, we present the application of this new assay centering on a scenario where *Pseudomonas aeruginosa* (*Pa*) encounters bile.

CF is a life-threatening, inherited, multi-organ disease, characterized by a universal thickening of a person’s mucosal layers, derived from mutations in the CF transmembrane conductance regulator (CFTR) gene [9,10]. Throughout their lives, people with CF (pwCF) are susceptible to chronic and ultimately deteriorating, persistent, polymicrobial lung infections, which account for over 90% of the morbidity and mortality associated with the disease [11]. *Pa* is arguably the most important bacterial pathogen among pwCF [12,13]. Once a pwCF is colonized with *Pa*, it is tough to eradicate, leading to adaptation and chronic infections in the majority of older children and adults [12,14]. Insights into how *Pa* finds its initial niches during early infection in pwCF could drive new clinical strategies to control or defeat this opportunistic pathogen.

One of the drivers of CF pulmonary infections is hypothesized to be aspirated bile, a consequence of gastroesophageal reflux disease (GERD) [15,16]. First described in 1975 [17], GERD is a major co-morbidity factor in pwCF, ranging between 35–81% with elevated prevalence since childhood (there is a lacking specific guideline for GERD in CF, bringing the underestimation into context without considering the asymptomatic/silent GERD cases) [15,16]. GERD-derived bile aspiration is associated with the early airway tract acquisition of *Pa* in CF patients [18]. Indeed, despite the inherent bactericidal nature of bile [19,20], *Pa* can tolerate it and even replicate in bile [21,22]. Additionally, bile stimulates *Pa* virulence, facilitates colonization, and further enhances infection through stimulating biofilm formation, Type VI Secretion, efflux pump expression, quorum sensing, and antibiotic tolerance, all of which are linked to the switch from an acute lifestyle to a persistent phase of infection [20,21,23,24,25]. To date, there is still a lack of knowledge on any chemotaxis behavior of *Pa* as a major CF pathogen toward GERD-derived bile, since most studies on bile chemotaxis have been limited to enteropathogenic bacteria, including *Vibrio cholerae*, *Campylobacter jejuni*, and *Salmonella* spp. [4,19,23,26]. We hypothesized that *Pa* would be attracted to bile and thus we used our new chemotaxis experimental system, which consists of human CFBE cells that mimic CF airway epithelia [6,7], as well as microcapillary assays to test the hypothesis.

## 2. Materials and Methods

### 2.1. Bacterial Strains and Growth Conditions

Biological replicates of *P. aeruginosa* PAO1 [27] were cultured in a minimal salts medium (MSB) supplemented with 27.5 mM of glucose, 0.5% (*w*/*v*) casamino acids (CAS) (obtained from Amresco at the highest purity commercially available), and 3% (*w*/*v*) bovine bile and incubated overnight at 37 °C. Secondary cultures were inoculated for 2 h at 37 °C in MSB with 3% bovine bile (the chemoattractant). Bile supplementation of overnight and secondary cultures was applied to provide *Pa* with time for metabolic adaptations. These growth conditions were used in all the experiments.

### 2.2. CFBE-Cell Preparation

CFBE cells (CFBE41o-), originally collected from a CF patient, are homozygous for the ΔF508-CFTR mutation, [6]. The CFBE cell line used in this study was a gift from Dr. J. P. Clancy, [28] as described previously [29]. The cells were maintained in Eagle’s minimal essential medium (MEM), supplemented with fetal bovine serum (FBS; 10% *v*/*v*), L-glutamine (2 mM), penicillin (50 U/mL), plasmocin (5 mg/mL), puromycin (2 mg/mL), and streptomycin (50 mg/mL), and placed in a 5% CO_2_/95% air incubator at 37 °C [7,29]. CFBE cells were grown at a seeding density of 2 × 10^6^ in glass-bottom Petri dishes (MatTek Corporation) for 7–9 days to establish confluent monolayers [29]. The MEM cell growth medium was switched to a modified one, known as “imaging medium” [29], on the day the experiment was carried out.

### 2.3. Chemotaxis Assays

#### 2.3.1. Chemoattractant Preparation

The chemoattractant (bovine bile) was prepared in chemotaxis buffer (CB; sodium phosphate buffer (50 mM; pH 7.0), disodium ethylenediaminetetraacetic acid (EDTA) (10 µM), and glycerol (0.05% (*w*/*v*)) [30]. To obtain physiology relevant concentrations [24,31], bile was diluted in CB in a series of 10-fold dilutions from 3% to 0.0003% (*w*/*v*). All studies were performed using bovine bile since human and bovine biles have similar compositions, but with different proportions of bile acids [32,33]. All bile solutions were mixed with 2% (*w*/*v*) low-melting-temperature-agarose (NuSieve GTG, Lonza) and were drawn into a 1 μL microcapillary tube in preparation for chemotaxis experiments.

#### 2.3.2. Qualitative Capillary Assay

The assay was performed as previously described by Parales and Harwood (2002) [34]. Briefly, a mid-exponential PAO1 pellet was harvested, washed once with CB and resuspended in aerated CB to a final OD_600_ = 0.1, and then located in a chamber made by a coverslip and a glass U-tube with prior verification of bacterial cells’ motility by microscopy. Microcapillaries, filled with each tested concentration of bile in a low-melting-temperature gel in CB, were inserted into the pool of bacteria. CAS and CB were included as positive or negative controls, respectively. The chemotactic response at the mouth of the capillaries was visualized at time points 0 and 5 min using dark-field microscopy at 4x magnification (an Olympus IX73 inverted microscope plus an Olympus TH4-100 halogen illuminator) and recorded using an Olympus DP73 CCD camera with the Olympus cellSens standard version 1.8 software. The dark-field illumination was generated by a Ph2 ring in the long-working distance condenser NA 0.55 using a UPlanFL N 4x NA 0.13 objective. Photographs were processed for contrast and brightness and centered using Adobe Photoshop Lightroom.

#### 2.3.3. Quantitative Capillary Assay

Quantitative capillary assays were performed as described previously [1,5]. Briefly, the capillary tubes were 1 μL disposable micropipettes sealed on one end using flame and filled with the chemoattractant bile resuspended in CB. *P. aeruginosa* PAO1 cultures were prepared as described above. PAO1 cells were harvested in the mid-exponential phase of growth (OD_660_ = 0.4–0.6) by centrifugation at 4600 revolutions per min (rpm) for 5 min, washed once with CB, and then bacterial cells were resuspended in CB to an OD_660_ of 0.1. PAO1 was pipetted into each U-tube, and 1 µL microcapillary tubes containing different bile concentrations (0.0003–3% *w*/*v*) were added to each U-tube. The assay was run for 30 min at room temperature, after which the microcapillaries were removed and their contents were serially diluted and plated to quantify colony forming units (CFU)s in capillaries. In all experiments, CB was included as a negative control and used for the normalization of the results.

#### 2.3.4. CFBE Cell-Bacteria Chemotaxis Assay

**Figure 1:**
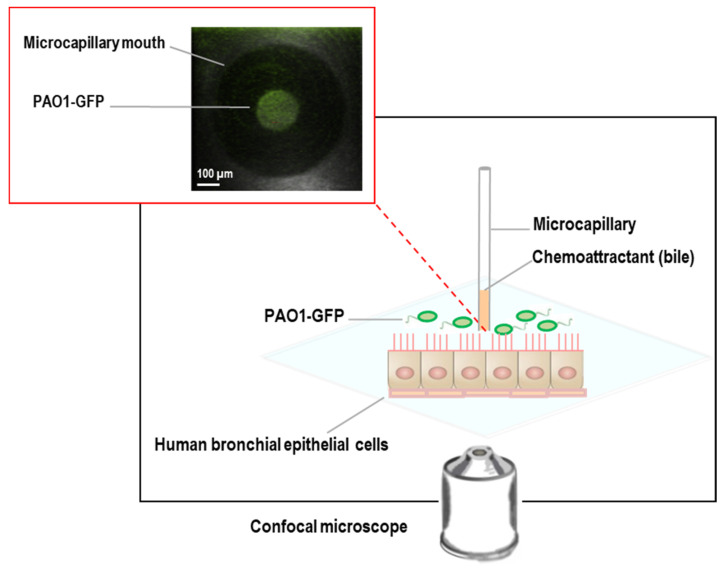
Human bronchial epithelial cell-bacteria chemotaxis system. The inset illustrates chemoattraction of green fluorescent protein (GFP)-labeled *P. aeruginosa* PAO1 (PAO1-GFP) cells towards bile.

In our experimental system (Figure 1), a monolayer culture of CFBE cells is grown on the bottom of a glass Petri dish above which sits a microcapillary tube containing bovine bile as the potential chemoattractant with physiologically relevant concentrations between 0.03% and 3% (*w*/*v*). Green fluorescent protein (GFP)-labeled *P. aeruginosa* PAO1 (PAO1-GFP) [35] cells were prepared, as described previously in Section 2.3.3, were introduced, and their movement was tracked over 10 min using confocal imaging. The capillary containing the bile component in a gel of 2% low-melting-temperature agarose was lowered into a suspension of motile PAO1-GFP and CFBE cells in a glass-bottom Petri dish closed with a lid in which a hole allowed the introduction of the capillary tubes, just to put the mouth in contact with the bacteria. GFP-expressing PAO1 motility was confirmed prior to the introduction using 0.3% (*w*/*v*) soft agar plates. The health and viability of CFBE cells in the presence of bile were confirmed using the LIVE/DEAD kit (Invitrogen; data not shown). In addition, to investigate the impact of any damage to the CFBE cells, we exposed the cells to −80 °C for 30 min and then 37 °C for 15 min as described in Text S1. The results confirmed that damage to the CFBE cells did not influence the migration of *Pa*, noting there was no difference between intact cells as well as damaged cells (Supplementary Text S1 and Figure S1).

#### 2.3.5. Replicates

Data are presented as the mean ± SE of at least five independent experiments, with three technical replicates each.

### 2.4. Microscopy

Confocal microscopy imaging was conducted on a Nikon TE2000 Live-Scan Swept Field Confocal microscope equipped with a QuantEM:512SCEMCCD camera (Photometrics, Tucson, AZ, USA), located in the Live Cell Imaging Core at the Dartmouth Lung Biology Center. In qualitative assays, confocal microscopy images were taken by the objective 10x during a time course between 0 and 10 min (time intervals of 2 min) in a system where a microcapillary containing the bile component in a gel of 2% low-melting-temperature agarose was lowered into a suspension of motile, PAO1-GFP and CFBE cells, as described before.

## 3. Results and Discussion

### 3.1. P. aeruginosa PAO1 Is Attracted toward Bovine Bile and Correlates with Bile Concentration

**Figure 2:**
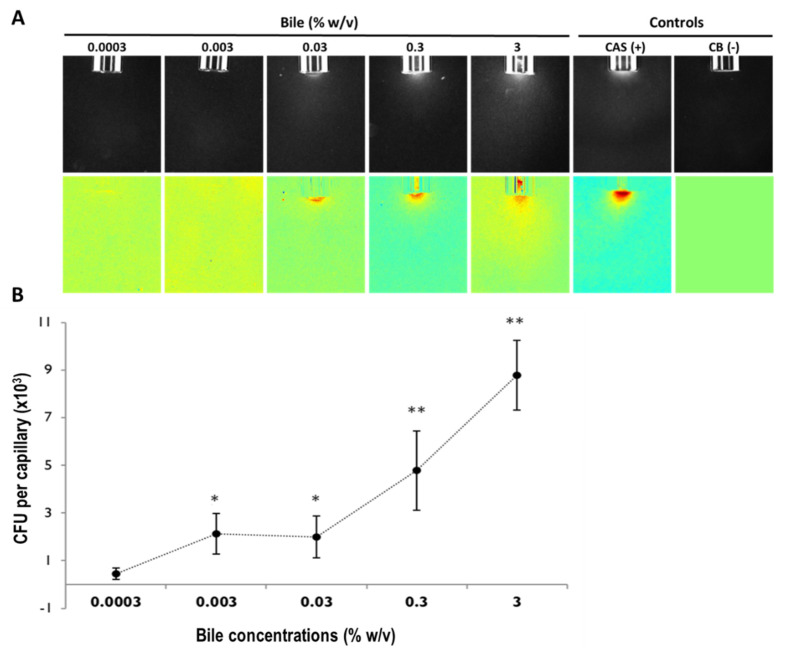
Chemotaxis of *P. aeruginosa* PAO1 toward bovine bile in the absence of cystic fibrosis -derived bronchial epithelial (CFBE) cells using qualitative (**A**) and quantitative (**B**) microcapillary assays. (**A**) PAO1 chemoattraction towards bile (0.0003–3% *w*/*v*). Top panel: Dark-field images of bacterial cells gathered at the mouth of microcapillaries containing attractants at time 5 min. Below panel: Color map “jet” (MATLAB R2013b version 8.2; www.Mathworks.com, accessed on 5 September 2013) representing a normalization with the time 0 min for each treatment. The positive control (casamino acids, (0.2%, *w*/*v*) (CAS)) and the negative control (chemotaxis buffer (CB)) are shown for contrast. (**B**) Quantitative assay illustrating the concentration-response curve of *P. aeruginosa* PAO1 to bovine bile (0.0003–0.3% *w*/*v*) diluted in chemotaxis buffer. Results are averaged of at least 18 capillaries from 7 independent experiments. The results have been normalized with CB. (Results with no normalization have been illustrated in Supplementary Figure S2). Error bars indicate standard error. A comparison between the five concentrations of bile tested and the negative control is indicated by the ANOVA test (* 0.01 < *p* ≤ 0.05; ** *p* ≤ 0.01).

First, we evaluated and confirmed *Pa* chemotaxis toward bile using the traditional qualitative (Figure 2A) and quantitative (Figure 2B, Supplementary Figure S2) microcapillary chemotaxis assays using a range of physiologically relevant bovine bile concentrations (0.0003–3% *w*/*v*) [24,31]. Casamino acids (CAS) and the chemotaxis buffer (CB) were used as the positive and negative controls, respectively. Figure 2 illustrates the PAO1 chemoattraction response toward bovine bile in a concentration-related manner.

### 3.2. PAO1 Is Attracted to Bovine Bile in the Novel CFBE Experimental System

The CFBE cell line was originally isolated from a pwCF (generated by Dr. Gruenert, UCSF) and subsequently was immortalized [6]. This cell line model recapitulates several aspects of CF lung disease, including the capability to form electrically-tight cell layers with functional cell-to-cell contacts, and has been used for gathering information about CF at the cellular level [7,36]. It was also used to elucidate mechanisms associated with *Pa* colonization, biofilm formation, and antimicrobial-agent efficacy evaluation [29,37].

**Figure 3:**
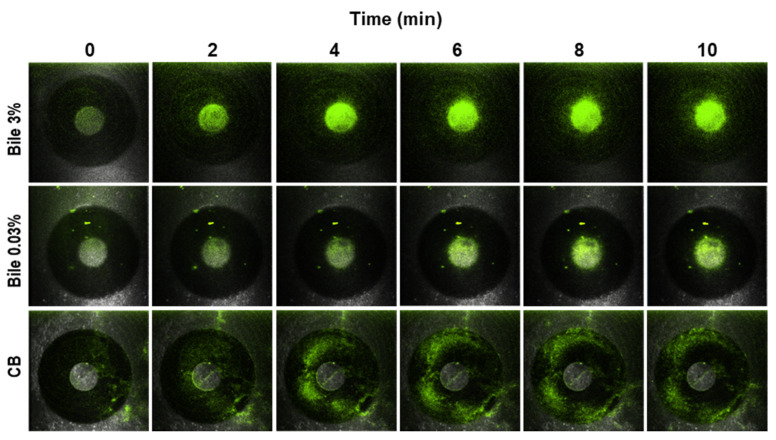
Chemotaxis of green fluorescent protein (GFP)-labeled *P. aeruginosa* PAO1 toward bovine bile using cystic fibrosis-derived bronchial epithelial (CFBE) cells. Confocal microscopy images (10×) of the chemoattraction of GFP-labeled PAO1 toward 0.03% and 3% (*w*/*v*) bovine bile on CFBE cells at times 0–10 min (two top panels). Chemotaxis buffer (CB) with no response is shown for contrast (below panel). PAO1 is attracted to bovine bile in an intact respiratory CFBE cell model, as illustrated in Figure 1.

First, to investigate whether the expression of GFP affected the chemotaxis response of PAO1, we tested the chemoattraction response of both the wild-type strain (no GFP) and GFP-labeled PAO1 toward 3% (*w*/*v*) bile in quantitative microcapillary assays as described before. Both strains exhibited the same chemoattraction, which was approximately fifty times higher in the presence of bile than the CB control (data not shown). We then evaluated the chemotaxis response of PAO1-GFP in the new chemotaxis assay. In a time-lapse experiment, PAO1-GFP can be seen (Figure 3) concentrating around the microcapillary mouth containing bovine bile, in a concentration- and time-dependent manner. By contrast, PAO1-GFP did not aggregate around the capillary mouth.

## 4. Conclusions

This experimental system demonstrates a new use for the CFBE cell culture system. Here, we show how *Pa* colonization in the CF lung may be preceded or enhanced by chemotactic responses to bile. We suggest that this model can facilitate studying *Pa*, and other motile CF opportunistic bacterial pathogens (e.g., *Burkholderia cepacia* complex and *Stenotrophomonas maltophilia*), chemotaxis, initial binding, as well as colonization at the same time [23,24,25,29,37]. In addition, this model could be useful for studies with eukaryotic cells, such as neutrophils, in the context of CF lung. Using this CF-derived model, which is more biologically relevant than the traditional standard assay systems, provides a closer insight into the chemoattraction of *Pa* toward bile in a biological condition that models the CF lung environment. This is a promising start and additional adjustments to this novel approach can be developed to closely represent the CF lung niche.

Here, our studies reveal that bile could be implicated in the accumulation of critical masses of *Pa* in the lung. Subsequent studies to assess the *Pa* chemoreceptor(s) involved in the PAO1 response, particularly toward individual constituents of bile [20,38], as well as an assessment of whether other *Pa* strains also exhibit chemoattraction, might cast additional light on *Pa* pathogenesis in the CF lung.

## Data Availability

Data are presented in the paper as well as the supporting information. Original data files will be available upon request from the authors.

## Supplementary Materials

The following are available online at https://www.mdpi.com/article/10.3390/microorganisms10040716/s1, Text S1, Damaged cystic fibrosis-derived bronchial epithelial (CFBE41o-, here CFBE) cells do not influence the chemotaxis of *P. aeruginosa* PAO1 toward bovine bile, Figure S1: Quantitative chemotaxis assay of *P. aeruginosa* PAO1 toward bile (3% *w*/*v*) in intact and damaged human cystic fibrosis bronchial epithelial (CFBE) cells, Figure S2: Quantitative assay illustrating non-normalized concentration-response of *P. aeruginosa* PAO1 to bovine bile (0.0003–0.3% *w*/*v*) diluted in chemotaxis buffer (CB). References [1,30] are cited in the Supplementary Materials.
