# Supplementary material for: Pseudomonas aeruginosa PAO1 Is Attracted to Bovine Bile in a Novel, Cystic Fibrosis-Derived Bronchial Epithelial Cell Model
Source: Microorganisms. 2022 Mar 26;10(4):716. doi: 10.3390/microorganisms10040716 (PMC9032244; doi:10.3390/microorganisms10040716)
Supplement: Supplementary file 1 [file microorganisms-10-00716-s001.zip › microorganisms-1612321-SI.pdf]

SUPPLEMENTAL MATERIAL

Communication

Pseudomonas aeruginosa PAO1 Is Attracted to Bovine Bile in a Novel, Cystic Fibrosis-Derived Bronchial Epithelial Cell Model

Shekooh Behroozian [1,†], Inmaculada Sampedro [2,3,†], Basanta Dhodary [1], Stephanie Her [4], Qianru Yu [4], Bruce A. Stanton [4] and Jane E. Hill [1,4,*]

1 Department of Chemical and Biological Engineering, University of British Columbia, 2360 E Mall, Vancouver, BC V6T 1Z3, Canada; shekooh@mail.ubc.ca (S.B.); basanta.dhodary@ubc.ca (B.D.)
2 Department of Microbiology, Faculty of Pharmacy, University of Granada, Campus de Cartuja s/n, 18071 Granada, GR, Spain; isampedro@ugr.es
3 Biomedical Research Center (CIBM), Biotechnology Institute, Avda del Conocimiento s/n, 18100 Armilla, Granada, GR, Spain
4 Thayer School of Engineering, Dartmouth College, Department of Microbiology and Immunology, Geisel School of Medicine at Dartmouth, Hanover, NH 03755, USA; stephanie.cindy.her@gmail.com (S.H.); qianru.yu@jax.org (Q.Y.); bruce.a.stanton@dartmouth.edu (B.A.S.)
* Correspondence: jane.hill@ubc.ca
† These authors contributed equally.

TEXT S1. Damaged cystic fibrosis -derived bronchial epithelial (CFBE41o-, here CFBE) cells do not influence the chemotaxis of *P. aeruginosa* PAO1 toward bovine bile.

Damaged CFBE cell preparation. To injure CFBE cells, the cells were suspended in chemotaxis buffer (CB) and then exposed to -80 °C for 30 min and then 37 °C for 15 min. Data are represented as the mean ± SEM of at least five independent experiments with three technical replicates each.

Quantitative chemotaxis with intact and damaged CFBE cells. Using our CFBE-chemotaxis system, the microcapillary tube containing bovine bile in 2% (*w*/*v*) agarose gel was incubated with *P. aeruginosa* PAO1 and CFBE cells for 30 min. Then, the capillary tube was removed and the colony forming units (CFU)s/capillary was determined by plating, as previously described [1,2].

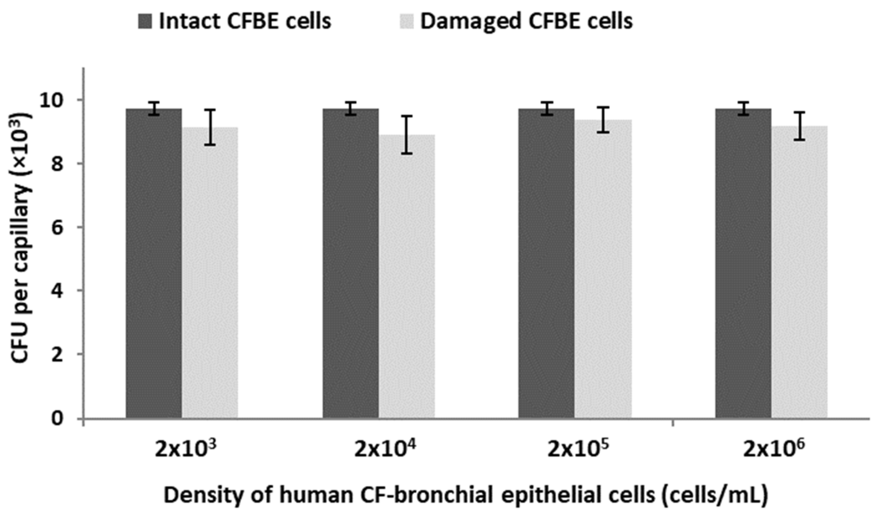

Figure S1. Quantitative chemotaxis assay of *P. aeruginosa* PAO1 toward bile (3% *w*/*v*) in intact and damaged human cystic fibrosis bronchial epithelial (CFBE) cells

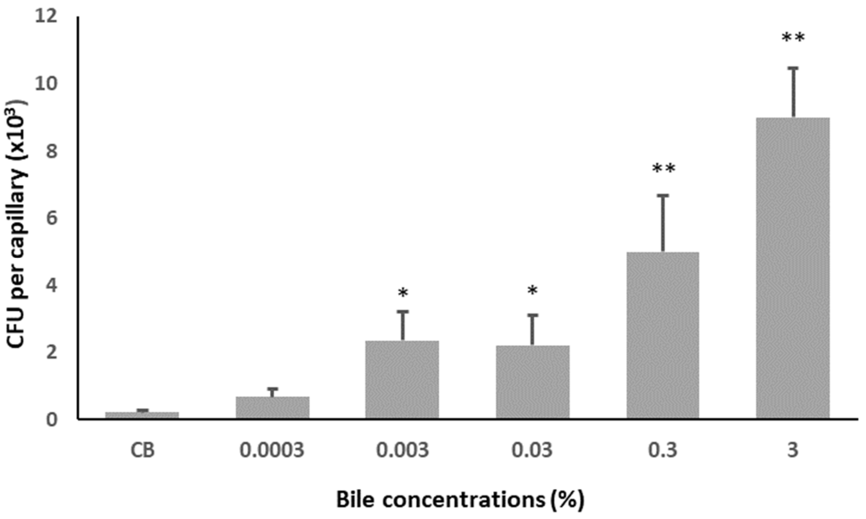

Figure S2. Quantitative assay illustrating non-normalized concentration-response of *P. aeruginosa* PAO1 to bovine bile (0.0003–0.3% *w*/*v*) diluted in chemotaxis buffer (CB). Error bars indicate standard error. Comparison between the five concentrations of bile tested and the negative control indicated by ANOVA test (* $0.01 < p \leq 0.05$; ** $p \leq 0.01$).

References

1. Adler, J. Chemotaxis in bacteria. *Science.* **1966**, *153(3737)*, 708–716. https://doi.org/10.1126/science.153.3737.708

2. Sampedro, I.; Kato, J.; Hill, J.E. Elastin degradation product isodesmosine is a chemoattractant for *Pseudomonas aeruginosa*. Microbiology (Reading). **2015**, *161(7)*, 1496–1503. https://doi.org/10.1099/mic.0.000090
